# A ribonuclease T2 protein FocRnt2 contributes to the virulence of *Fusarium oxysporum* f. sp. *cubense* tropical race 4

**DOI:** 10.1111/mpp.13502

**Published:** 2024-08-08

**Authors:** Yanqiu He, Pengfei Li, Xiaoshu Zhou, Shaukat Ali, Jie Zhu, Yini Ma, Jieling Li, Nan Zhang, Huaping Li, Yunfeng Li, Yanfang Nie

**Affiliations:** ^1^ Guangdong Province Key Laboratory of Microbial Signals and Disease Control, College of Plant Protection South China Agricultural University Guangzhou China; ^2^ Institute of Plant Protection and Agro‐Products Safety Anhui Academy of Agricultural Sciences Hefei China; ^3^ College of Materials and Energy South China Agricultural University Guangzhou China

**Keywords:** effector, *Fusarium oxysporum* f. sp. *cubense*, ribonucleases T2, transient expression, virulence

## Abstract

Banana Fusarium wilt, caused by *Fusarium oxysporum* f. sp. *cubense* tropical race 4 (Foc TR4), is a major disease of banana plants worldwide. Effector proteins play critical roles in banana–Foc TR4 interaction. Our previous studies highlighted a ribonuclease protein belonging to the T2 family (named as FocRnt2) in the Foc TR4 secretome, which was predicted to be an effector. However, its biological function in Foc TR4 infection is still unclear. Herein, we observed significant expression of *FocRnt2* during the early stage of fungal infection in planta. A yeast signal sequence trap assay showed that FocRnt2 contained a functional signal peptide for secretion. FocRnt2 possessed ribonuclease activity that could degrade the banana total RNA in vitro. Subcellular localization showed that FocRnt2 was localized in the nucleus and cytoplasm of *Nicotiana benthamiana* leaves. Transient expression of *FocRnt2* suppressed the expression of salicylic acid‐ and jasmonic acid‐signalling marker genes, reactive oxygen species accumulation, and BAX‐mediated cell death in *N*. *benthamiana*. *FocRnt2* deletion limited fungal penetration, reduced fusaric acid biosynthesis in Foc TR4, and attenuated fungal virulence against banana plants, but had little effect on Foc TR4 growth and sensitivity to various stresses. Furthermore, *FocRnt2* deletion mutants induced higher expression of the defence‐related genes in banana plants. These results suggest that FocRnt2 plays an important role in full virulence of Foc TR4, further improving our understanding of effector‐mediated Foc TR4 pathogenesis.

## Graphical abstract

FocRnt2, a novel effector from *Fusarium oxysporum* f. sp. *cubense*, regulates fusaric acid biosynthesis, promotes fungal infection, suppresses plant immune responses, and contributes to fungal virulence.

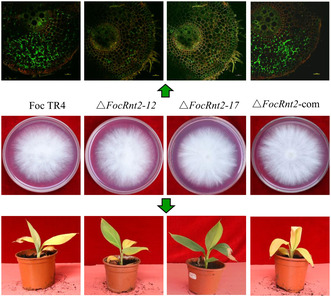

## 1 INTRODUCTION

The ascomycete fungus *Fusarium oxysporum* f. sp. *cubense* (Foc) causes banana Fusarium wilt, which is a devastating vascular wilt disease of banana plants (Bubici et al., 2019). Foc has been classified into three physiological races based on their host range and pathogenicity, named as race 1 (Foc1), race 2 (Foc2), and race 4 (Foc4) (Thangavelu et al., 2021). According to regional distribution and temperature adaptation ability, Foc4 has been further divided into tropical race 4 (Foc TR4) and subtropical race 4 (Foc STR4) (Ploetz, 2015). Foc TR4 is known as the most virulent strain, which can attack almost all banana cultivars in China (Guo et al., 2015).

During plant–pathogen co‐evolution, pathogenic fungi use different strategies to colonize plants, obtain nutrients, and cause disease (van der Does & Rep, 2017). Most phytopathogenic fungi can secrete a variety of proteins at different infection stages to manipulate the host and facilitate fungal colonization (Pandey et al., 2018). Among these secreted proteins, effectors play essential roles during fungal‐–plant interactions by facilitating infection or triggering defence responses in host plants (Deng et al., 2017; Tanaka et al., 2015; Vincent et al., 2020). During the infection process, Foc produces diverse effectors to modify the cell structure and metabolic pathways of banana plants (Czislowski et al., 2018; Zhao et al., 2020). Several Foc TR4 effectors, such as SIX proteins (Secreted In Xylem), FoCupin1, cerato‐platanin, M35 family metalloproteinases, FTF1, and OASTL, have been reported to play an important role in fungal penetration, infection, and full virulence (An et al., 2019; Liu et al., 2019; Lorrain et al., 2015; Wang et al., 2020; Yan et al., 2022; Zhang et al., 2021).

Ribonucleases (RNases), a class of enzymes that can hydrolyse RNA phosphodiester bonds, can be divided into three families (RNase T1, RNase A, and RNase T2) according to the characteristics of protein structure, function, and optimal pH (Wu et al., 2020). RNase T2 is a family of endoribonucleases without absolute base specificity and has two types (intracellular RNase T2 and secreted RNase T2) (Acquati et al., 2011). Previous studies showed that RNase T2 secreted by many pathogens plays crucial roles during plant–pathogen interactions (Lu et al., 2018; Qian et al., 2022). In some pathogenic fungi, secreted RNase T2 is considered to be a new type of effector that inhibits plant resistance responses against fungal infection (Keller, 2019; Pennington et al., 2019). Qian et al. (2022) reported that the RNase T2 protein from *F*. *oxysporum* f. sp. *lycopersici* is able to degrade the host plant's total RNA in vitro, and deletion of the *FoRnt2* gene reduces fungal virulence. Deletion of two RNase T2 genes, *nuc1* and *nuc2*, from *Ustilago maydis* reduces fungal pathogenicity and significantly delays the completion of the pathogenic lifecycle (Mukherjee et al., 2020). Although these reports indicate that RNase T2 proteins are involved in some fungal virulence, the underlying mechanisms of RNase T2 in Foc TR4 still require further elucidation.

In our previous study, a shotgun‐based secretome analysis of Foc TR4 was conducted, and 70 candidate effectors were predicted using bioinformatic approaches (He et al., 2021). Here, we characterized a candidate effector, the RNase T2 protein (named FocRnt2) from the Foc TR4 secretome that belongs to the RNase T2 family, using *Agrobacterium*‐mediated transient expression in *Nicotiana benthamiana*. The FocRnt2 protein is a classical secreted protein with a N‐terminal signal peptide without any transmembrane domain or glycosylphosphatidylinositol (GPI)‐anchor site. Sequence analysis indicated that FocRnt2 and its homologues are evolutionarily conserved in phytopathogenic fungi. *FocRnt2* deletion dramatically reduced the virulence of Foc TR4 and suppressed the defence responses in banana plantlets. FocRnt2 also suppressed Bcl2‐associated X protein (BAX)‐induced cell death and reactive oxygen species (ROS) accumulation in *N. benthamiana*. Our results indicated that FocRnt2 might contribute to fungal virulence during the Foc TR4–banana interaction.

## 2 RESULTS

### 2.1 *FocRnt2* is highly conserved among different *Fusarium* strains

**FIGURE 1:**
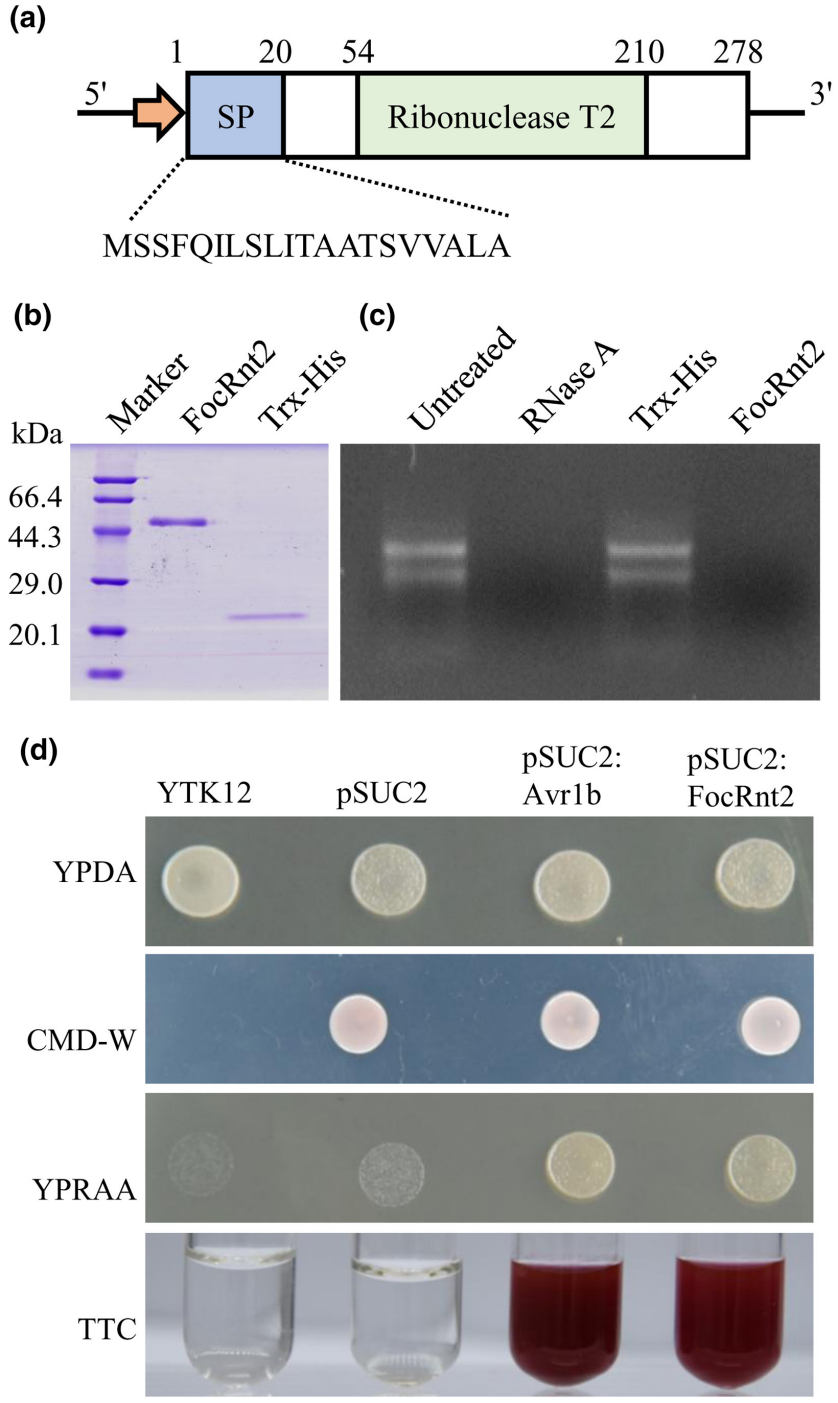
FocRnt2 protein has RNase activity and secretion function. (a) Protein structure diagram. The signal peptide (SP) and the ribonuclease domain were predicted by SignalP v. 5.0 and Pfam, respectively. (b) SDS‐PAGE analysis of the recombinant protein Trx‐His‐FocRnt2 (FocRnt2) and the tag protein Trx‐His, which were expressed in *Escherichia coli* Rosetta (DE3). (c) RNase activity assay was performed using the purified FocRnt2 protein by incubation with total RNA from banana roots. RNase A was used as a positive control, while the tagged protein Trx‐His was used as a negative control. Untreated total RNA without any treatments. (d) Function validation of the signal peptide of FocRnt2. The yeast YTK12 strain carrying the FocRnt2 SP sequence fused in the pSUC2 vector (pSUC2:FocRnt2) was able to grow in the CMD−W and YPRAA media and also induced a red colour reaction. The YTK12 strain and YTK12 carrying the empty pSUC2 vector served as negative controls. The YTK12 carrying pSUC2:Avr1b was used as a positive control. The invertase activity was confirmed by the reduction of 2,3,5‐triphenyltetrazolium chloride (TTC) to red triphenylformazan.

Our previous secretome analysis of Foc TR4 showed that the RNase T2 protein (named FocRnt2) was characterized as a candidate effector. The FocRnt2 protein is encoded by the *FOIG_10760* gene and contains 278 amino acids with an RNase T2 domain (Figure 1a). FocRnt2 was predicted to be a classically secreted extracellular protein with an N‐terminal signal peptide, without transmembrane domain or GPI anchoring signal. BLAST searches against the NCBI protein database showed that RNase T2 is widely present in plant‐pathogenic fungi. Phylogenetic analysis showed that FocRnt2 has a high degree of similarity with several proteins of fungal plant pathogens, such as ribonuclease Trv (ENH66201.1; similarity: 97.48%) from *F*. *oxysporum* f. sp. *cubense* race 1, uncharacterized protein (KNB09966.1; similarity: 98.92%) from *F*. *oxysporum* f. sp. *lycopersici*, ribonuclease T2 (KAF5596200.1; similarity: 93.53%) from *F*. *pseudocircinatum* (Figure S1). Moreover, a BLASTP search against the NCBI NR database also identified a FocRnt2 homologue encoded by the *FOIG‐14337* gene in Foc TR4, although the levels of amino acid sequence similarity were considerably lower (35.3%).

### 2.2 FocRnt2 has RNase activity and contains a signal peptide with secretion function

To confirm the RNase activity of FocRnt2, we expressed and purified FocRnt2 fusion protein with a Trx‐His tag in *Escherichia coli* Rosetta (DE3) (Figure 1b). The RNase activity of the FocRnt2 recombinant proteins was tested by incubation with total RNA from banana roots. Both FocRnt2 and RNase A (as a positive control) could significantly degrade the total RNA, while the Trx‐His tag protein (as a negative control) could not degrade the RNA under the same conditions (Figure 1c). These results demonstrated that FocRnt2 possessed RNase activity.

To further validate the secretion function of the predicted signal peptide, the SP sequence of FocRnt2 (amino acids 1–20) was cloned into the pSUC2 vector. All yeast YTK12 strains could grow on YPDA plates. The yeast strains harbouring the pSUC2 vector could grow on CMD−W plates. However, only the strains containing pSUC2:FocRnt2 or pSUC2:Avr1b could grow on YPRAA plates and enabled the catalysis of 2,3,5‐triphenyltetrazolium chloride (TTC) to generate the insoluble red‐coloured triphenylformazan (Figure 1d). In contrast, YTK12 and the strain carrying the empty pSUC2 vector used as a negative control could not grow on YPRAA plates or change the colour of the culture (Figure 1d). These results suggested that FocRnt2 carries a functional secretory signal peptide and could be secreted from Foc TR4.

### 2.3 *FocRnt2* is highly expressed during the early stages of fungal infection

**FIGURE 2:**
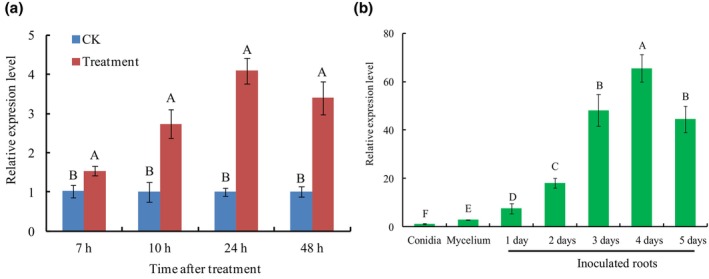
Reverse transcription‐quantitative PCR analysis of *FocRnt2* expression. (a) Expression of the *FocRnt2* gene in *Fusarium oxysporum* f. sp. *cubense* tropical race 4 (Foc TR4) conidia cultured in NCM medium (CK, control) or NCM medium plus banana plant extracts (Treatment). (b) Expression of the *FocRnt2* gene at different developmental and infective stages. *FoEF1a* was used as the internal reference gene. Values are the means based on three independent experiments, and bars indicate standard deviations. Different letters indicate statistical significance (*p* < 0.05) using Duncan's new multiple range method.

To investigate the expression levels of *FocRnt2* in Foc TR4, reverse transcription‐quantitative PCR (RT‐qPCR) analysis was performed using fresh fungal conidia cultured in NCM medium plus banana root extracts to mimic the banana–Foc TR4 interaction (He et al., 2021). The expression levels of *FocRnt2* were significantly up‐regulated and peaked at 24 h post‐induction (Figure 2a). To examine whether the expression of *FocRnt2* varied at different developmental and infection stages of Foc TR4, RT‐qPCR assays were carried out from fungal conidia, mycelia, and infected banana roots after inoculation with Foc TR4 conidia. *FocRnt2* was strongly induced during the early stage of Foc TR4 infection and peaked at 4 days (Figure 2b). However, the expression of *FocRnt2* in fungal conidia or mycelia was remarkably lower than those in the infection stages. These results indicate that *FocRnt2* can be highly induced after induction or in the early stage of fungal infection, suggesting that *FocRnt2* may be crucial for Foc TR4 penetration and infection.

### 2.4 FocRnt2 inhibits plant immune responses in *N*. *benthamiana*

**FIGURE 3:**
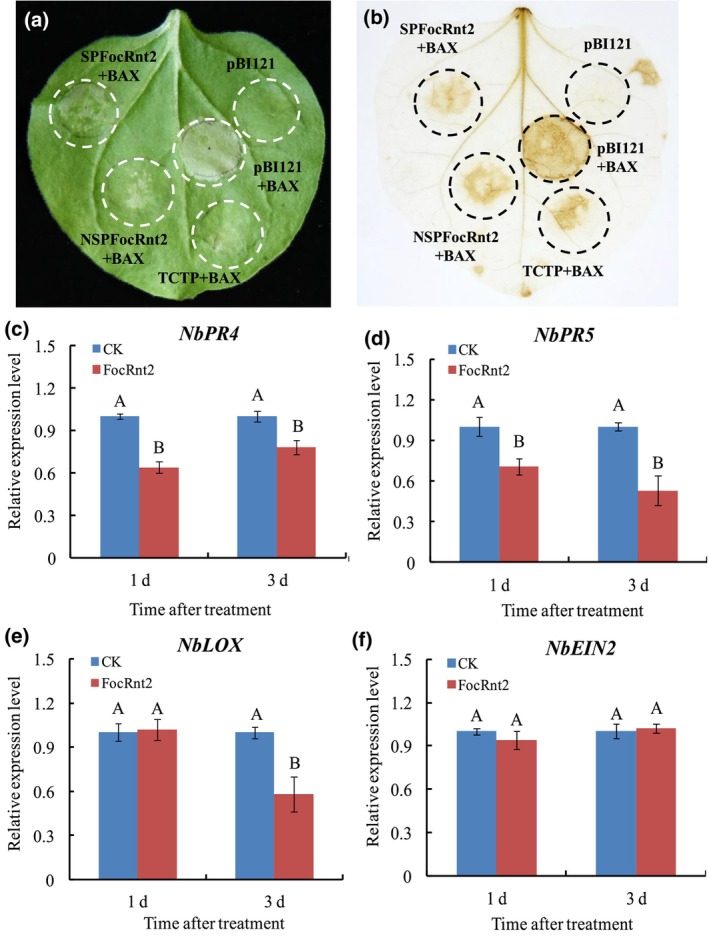
FocRnt2 suppresses plant immune responses in *Nicotiana benthamiana*. (a) FocRnt2 could inhibit BAX‐induced cell death in *N*. *benthamiana* leaves. *N*. *benthamiana* leaves were infiltrated with *Agrobacterium tumefaciens* expressing SPFocRnt2 (with signal peptide), NSPFocRnt2 (without signal peptide), pBI121 empty vector (as negative control), or TCTP (translationally controlled tumour protein) as positive control) 2 days before infiltration with *A*. *tumefaciens* expressing BAX. (b) Reactive oxygen species (ROS) accumulation in (a) was detected by 3,3′‐diaminobenzidine (DAB) staining. The images were recorded 4 days after infiltration. (c–f), Transcription patterns of four defence‐related genes, *NbPR4* (c) *NbPR5* (d), *NbLOX* (e), and *NbEIN2* (f) in *N*. *benthamiana* after infiltration with *A*. *tumefaciens* expressing FocRnt2 or pBI121 empty vector (CK) as determined by reverse transcription‐quantitative PCR. Values are the means based on three independent experiments, and bars indicate standard deviations. Different letters indicate statistical significance (*p* < 0.05) using Duncan's new multiple range method.

*SPFocRnt2* (*FocRnt2* with signal peptide) or *NSPFocRnt2* (*FocRnt2* without signal peptide) were separately constructed into the pBI121 vector and transiently expressed in *N*. *benthamiana* for further functional characterization of FocRnt2. Both SPFocRnt2 and NSPFocRnt2 could inhibit BAX‐induced cell death in *N*. *benthamiana* leaves at 3 days post‐infiltration (Figure 3a); however, neither of them was able to induce the cell death (Figure S2a). ROS accumulation was detected by 3,3′‐diaminobenzidine (DAB) staining. The results showed that SPFocRnt2 and NSPFocRnt2 suppressed BAX‐mediated ROS accumulation (Figure 3b) without inducing ROS accumulation in *N*. *benthamiana* (Figure S2b). These observations were further confirmed by measuring the expression levels of four defence‐related genes using RT‐qPCR after infiltration with FocRnt2. The expression of the salicylic acid (SA) signalling marker genes *NbPR4* and *NbPR5* and the jasmonic acid (JA) signalling marker gene *NbLOX* (Wang et al., 2021) were significantly decreased (Figure 3c–e); however, the expression of the ethylene signalling marker gene *NbEIN2* (Zhang et al., 2010) showed no significant changes in *N*. *benthamiana* leaves (Figure 3f). Taken together, the transient expression of *FocRnt2* suppressed the plant immune responses by weakening the SA‐ and JA‐mediated defence response, thus promoting fungal infection.

### 2.5 FocRnt2 is localized in the nucleus and cytoplasm in *N*. *benthamiana*

**FIGURE 4:**
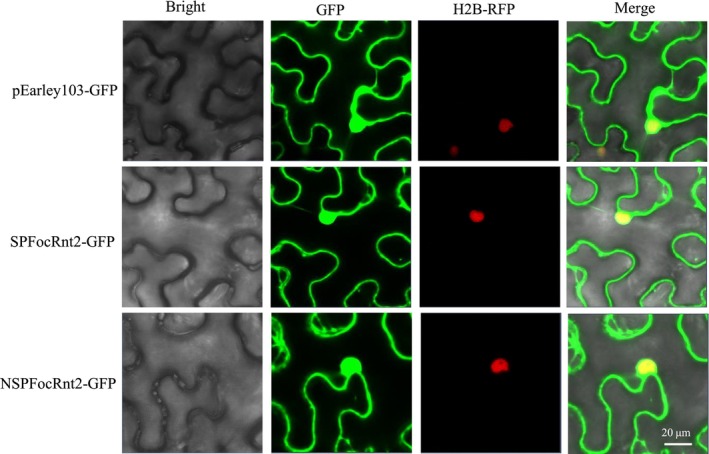
Subcellular localization of FocRnt2 in *Nicotiana benthamiana* leaves. Fluorescence micrographs showed the subcellular distribution of GFP, GFP‐SPFocRnt2, and GFP‐NSPFocRnt2 in H2B‐RFP transgenic *N*. *benthamiana* at 2 days post‐agroinfiltration. pEarley‐GFP was used as the negative control. H2B‐RFP was used to visualize the nucleus. Scale bars represent 20 μm.

To investigate FocRnt2 localization in plant cells, FocRnt2 with or without the signal peptide was separately fused to green fluorescent protein (GFP) in pEarly103 and transiently expressed in H2B‐RFP transgenic *N*. *benthamiana* leaves using *A*. *tumefaciens*‐mediated transformation system. At 2 days post‐agroinfiltration, fluorescence microscopy detected GFP fluorescence for the control vector encoding GFP alone in the nucleus and cytoplasm, while H2B‐RFP showed RFP signals in the nucleus. Similarly, GFP fluorescence for FocRnt2‐GFP and NSPFocRnt2‐GFP was detected in the cytoplasm as well as in the nucleus of *N*. *benthamiana* cells (Figure 4), suggesting that FocRnt2 has a dual‐localization in plant cells.

### 2.6 FocRnt2 has no effect on hyphal growth and conidiation

**FIGURE 5:**
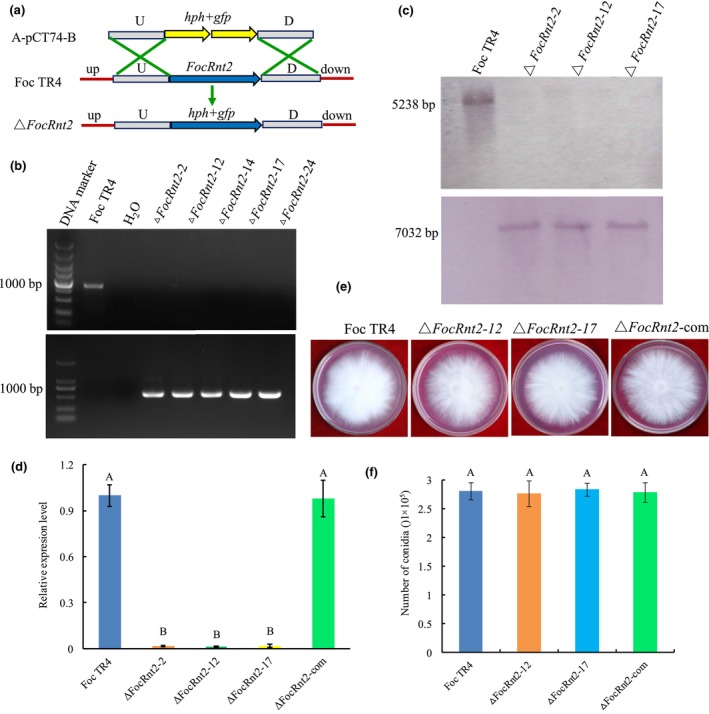
Generation of *FocRnt2* deletion and complementation mutants of *Fusarium oxysporum* f. sp. *cubense* tropical race 4 (Foc TR4). (a) Strategy for *FocRnt2* gene deletion by homologous recombination. U, upstream flanking region; D, downstream flanking region. (b) PCR confirmation using primers for *FocRnt2* (upper) and *hph* (lower). (c) Southern blot analysis using *FocRnt2* (upper) and *hph* (lower) as probes. (d) Reverse transcription‐quantitative PCR detection of *FocRnt2* expression in three deletion mutants and a complementation strain. (e) Colony morphologies on potato dextrose agar. Photographs were taken after incubation for 6 days. (f) Conidiation. Foc TR4, the wild‐type strain; Δ*FocRnt2*‐12 and Δ*FocRnt2*‐17, *FocRnt2* deletion mutants; Δ*FocRnt2*‐com, *FocRnt2* complementation strain. Values are the means based on three independent experiments, and bars indicate standard deviations. Different letters indicate statistical significance (*p* < 0.05) using Duncan's new multiple range method.

To investigate the role of FocRnt2 in Foc TR4, the homologous recombination method was used to delete the *FocRnt2* gene in the Foc TR4 wild‐type (WT) strain (Figure 5a). A total of 25 mutants were confirmed to lack the *FocRnt2* gene and contain the *hph* gene by PCR from 38 hygromycin‐resistant transformants (Figure 5b). Three mutants (Δ*FocRnt2*‐2, Δ*FocRnt2*‐12, and Δ*FocRnt2*‐17) were further validated by Southern blot analysis using a *FocRnt2*‐specific probe and a *hph*‐specific probe and by RT‐qPCR, which showed that these mutants lacked *FocRnt2* (Figure 5c,d). The three deletion mutants showed no morphological differences on potato dextrose agar (PDA), minimal medium (MM), and complete medium (CM), having growth rate, colony morphology, and conidia highly similar to the WT (Figure 5f; Figure S3). Thus, Δ*FocRnt2*‐12 and Δ*FocRnt2*‐17 were randomly selected as representative mutants for further analyses. In addition, the *FocRnt2* gene was introduced into the Δ*FocRnt2*‐12 strain to obtain the complementation strains (Δ*FocRnt2*‐12‐3‐com, Δ*FocRnt2*‐12‐8‐com, Δ*FocRnt2*‐12‐17‐com, Δ*FocRnt2*‐12‐19‐com, and Δ*FocRnt2*‐12‐26‐com), which was confirmed by PCR (Figure S4a) and RT‐qPCR (Figure 5d; Figure S4b). No difference in the growth rate, colony morphology, or conidia was observed between the WT strain and these complementation strains (Figure 5f; Figure S3); thus, a complementation strain Δ*FocRnt2*‐12‐3‐com (named as Δ*FocRnt2*‐com) was randomly selected for further analyses. These results suggested that FocRnt2 was unnecessary for fungal growth and conidiation.

### 2.7 FocRnt2 has no effect on sensitivity to various stresses

To evaluate the function of FocRnt2 related to stress responses, the tolerance to oxidative, osmotic, and cell wall integrity stress (including H_2_O_2_, NaCl, sorbitol, SDS, Congo red [CR], or calcofluor white [CFW]) was determined by culturing the fungal strains on PDA supplemented with different chemicals. As shown in Figure S5, no significant differences were observed in colony morphology and the growth rate among the WT, *FocRnt2* deletion mutants, and the complementation strains under any of the stresses on PDA. These results implied that FocRnt2 is not necessary for various stress responses.

### 2.8 FocRnt2 is necessary for fungal virulence

**FIGURE 6:**
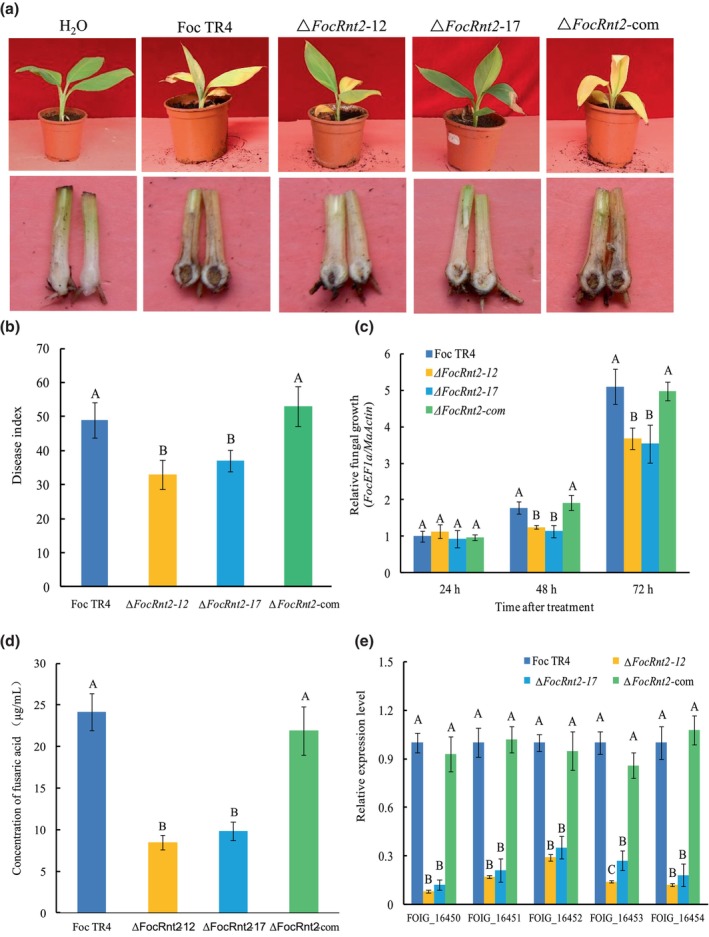
*FocRnt2* deletion attenuated fungal virulence and reduced fusaric acid content in *Fusarium oxysporum* f. sp. *cubense* tropical race 4 (Foc TR4). (a) Disease symptoms on banana cultivar cv. Brazilian 30 days post‐inoculation. (b) Disease index. (c) Relative fungal biomass assay in the infected roots. The relative fungal growth was measured by [2^[*C*t(*MaActin*)−*C*t(*FoEF1a*)]^ × 100] as determined by quantitative PCR. Values are the means from three independent experiments, and bars indicate standard deviations. The letters above the bars indicate a significant difference at 0.05 level using Duncan's multiple range test. (d) Fusaric acid contents of the wild type (WT), *FocRnt2* deletion mutants, and complemented strain cultured in Czapek Dox medium supplemented with banana extracts. (e) The expression of five fusaric acid biosynthetic genes in different strains as determined by reverse transcription‐quantitative PCR. Values are the means from three independent experiments, and bars indicate standard deviations. The letters above the bars indicate a significant difference at 0.05 level using Duncan's multiple range test.

To investigate whether *FocRnt2* is involved in Foc TR4 virulence, the susceptible banana cultivar Brazilian was inoculated with fresh conidial suspensions of WT, Δ*FocRnt2*‐12, Δ*FocRnt2*‐17, and Δ*FocRnt2*‐com. The *FocRnt2* deletion mutants resulted in reduced discolouration of the leaves, vascular tissue, and pseudostem, while the WT and Δ*FocRnt2*‐com caused more typical symptoms, such as yellow leaves and reddish‐brown pseudostem, in banana seedlings (Figure 6a). An obvious delay in disease symptom progression was also observed in *FocRnt2* deletion mutant‐inoculated banana plants. Consistent with the symptom observation, the disease index of *FocRnt2* deletion mutant‐inoculated banana plants was significantly lower than those of WT‐ and Δ*FocRnt2*‐com‐inoculated plants (Figure 6b). To further evaluate whether the *FocRnt2* deletion affected the fungal growth in planta, we assessed the relative fungal biomass in the infected roots by quantitative PCR (qPCR). The biomass of *FocRnt2* deletion mutants in banana roots was markedly lower than that of the WT and Δ*FocRnt2*‐com strains (Figure 6c). These results showed that *FocRnt2* deletion resulted in reduced virulence of Foc TR4 to banana, confirming the critical role of *FocRnt2* in Foc TR4 virulence.

### 2.9 FocRnt2 is required for fusaric acid biosynthesis

Fusaric acid (FA) is the main toxin of Foc TR4, which plays an important role in fungal virulence (Liu et al., 2020; Niehaus et al., 2014). As *FocRnt2* is required for Foc TR4 virulence, we measured FA production in *FocRnt2* deletion mutants by culturing the spores in Czapek Dox (CD) medium supplemented with banana extracts to mimic the banana–Foc TR4 interaction (Yan et al., 2022). Compared with the WT and Δ*FocRnt2*‐com strains, FA content was significantly reduced in Δ*FocRnt2*‐12 (Figure 6d). We further examined the expression patterns of the FA biosynthetic genes *FOIG_16450*, *FOIG_16451*, *FOIG_16452*, *FOIG_16453*, and *FOIG_16454*. Consistent with our results of FA content, the expression of all the five FA biosynthetic genes was significantly down‐regulated in *FocRnt2* deletion mutants (Figure 6e). These results indicated that *FocRnt2* resulted in the reduction of FA production, thus attenuating fungal virulence.

### 2.10 FocRnt2 promotes Foc TR4 infection in banana plants

**FIGURE 7:**
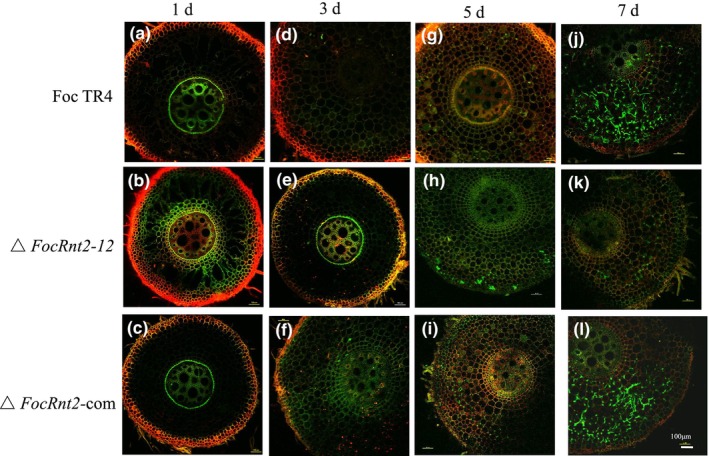
Colonization and expansion in banana roots of cultivar Brazilian infected with GFP‐tagged *Fusarium oxysporum* f. sp. *cubense* tropical race 4 (Foc TR4), *FocRnt2* deletion mutant, and Δ*FocRnt2*‐com complemented strain at different time points after inoculation. (a–c) 1 day post‐inoculation (dpi); (d–f) 3 dpi; (g–i) 5 dpi; (j–l) 7 dpi. Scale bars represent 100 μm.

To determine whether the reduced virulence of the *FocRnt2* deletion mutant was due to an infection defect, histopathological observation of the fungal infection process was monitored. The roots of banana plants inoculated with GFP‐expressing Foc TR4, *FocRnt2* deletion mutants, and the complemented strain were sampled at various time points. Confocal laser‐scanning microscopy observation showed that all the strains could penetrate the vascular bundle tissues of the banana roots. However, there were some differences in the infection process between *FocRnt2* deletion mutants and the WT and complemented strains. The conidia of the strains attached to the roots at 1 day post‐infection (dpi) (Figure 7a–c). At 3 dpi, most of the spores germinated and developed into hyphae (Figure 7d–f), and a small amount of hyphae of the WT and Δ*FocRnt2*‐com started to grow along the epidermal cell gaps (Figure 7d,f). At 5 dpi, the hyphae of Δ*FocRnt2* started to penetrate the outer epidermal cells of the roots (Figure 7h), whereas the hyphae of Foc TR4 and Δ*FocRnt2*‐com penetrated the parenchyma cells of the root epidermal cells (Figure 7g,i), following the cell gap expansion of the parenchyma cells. At 7 dpi, the hyphae of Δ*FocRnt2* gradually penetrated the epidermal cells and grew along the intercellular spaces of the inner parenchyma cells (Figure 7k), whereas the hyphae of FocTR4 and Δ*FocRnt2*‐com started to expand longitudinally along the intercellular spaces of the phloem (Figure 7j,l) and a small portion of the hyphae entered the catheter of the vascular bundle. These results indicated that the colonization and expansion of Δ*FocRnt2* was apparently slower than that of the WT and Δ*FocRnt2*‐com at the same time points in the infected roots.

### 2.11 FocRnt2 suppresses plant immunity responses in banana plants

**FIGURE 8:**
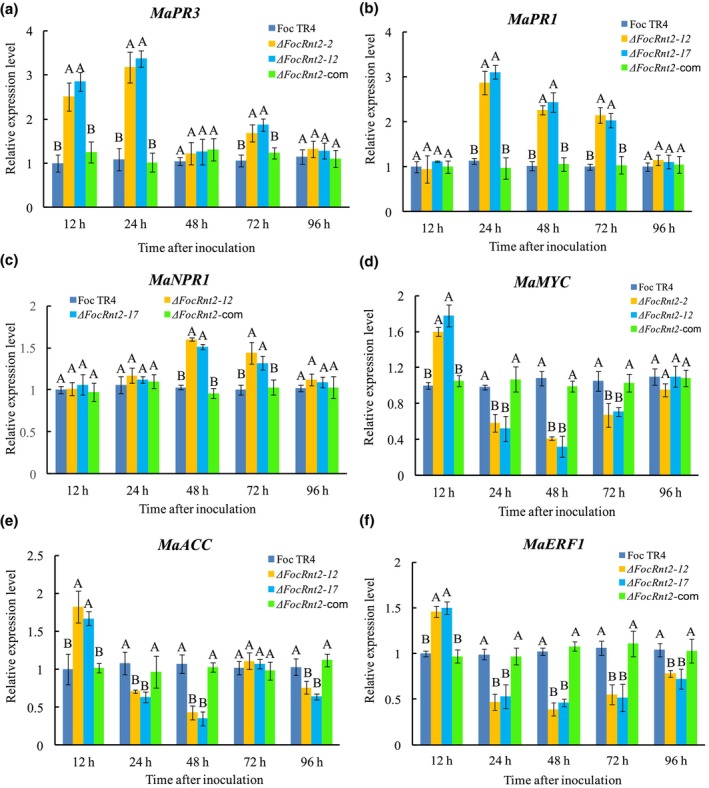
Transcription patterns of the defence‐related genes in banana cultivar Brazilian after inoculation with the wild type (WT), *FocRnt2* deletion mutants, and complemented strain as determined by reverse transcription‐quantitative PCR. (a) *MaPR3*; (b) *MaPR1*; (c) *MaNPR1*; (d) *MaMYC*; (e) *MaACO*; (f) *MaACC*. *Fusarium oxysporum* f. sp. *cubense* tropical race 4 (Foc TR4), the wild‐type strain; Δ*FocRnt2*‐12 and Δ*FocRnt2*‐17, *FocRnt2* deletion mutants; Δ*FocRnt2*‐com, *FocRnt2* complementation strain. Values are the means based on three independent experiments, and bars indicate standard deviations. The letters above the bars indicate a significant difference at 0.05 level using Duncan's multiple range test.

To determine whether the attenuated virulence of Δ*FocRnt2* is related to the plant defence response, we measured the expression of the marker genes for the SA signalling pathway (*MaPR1*, *MaPR3*, and *MaNPR1*) and JA/ethylene signalling pathway (*MaMYC*, *MaERF1*, and *MaACC*) (Cheng et al., 2015). The expression of SA‐signalling marker gene *MaPR3* was significantly induced at 12, 24, and 72 h after inoculation with *FocRnt2* deletion mutants (Figure 8a); *MaPR1* increased at 24, 48, and 72 h (Figure 8b); *MaNPR1* was up‐regulated at 48 and 72 h after inoculation (Figure 8c) compared with those of the WT‐ and Δ*FocRnt2*‐com‐inoculated controls. The JA/ET‐signalling marker genes *MaMYC*, *MaERF1*, and *MaACC* were remarkably increased at 12 h but decreased at 24, 48, 72, or 96 h after inoculation with *FocRnt2* deletion mutants (Figure 8d–f). These results suggested that the deletion of *FocRnt2* may attenuate the suppression of multiple banana defence responses during the early stage of Foc TR4 infection, thus limiting Foc TR4 penetration and infection.

## 3 DISCUSSION

RNases represent one of the most important groups of enzymes in life and participate in a variety of cellular processes, including replication, transcription, cell death, and immunity (Mukherjee et al., 2020). RNase T2 is one of the three earliest identified RNases (i.e., RNase T1, RNase A, and RNase T2), and is widely distributed in viruses, bacteria, fungi, plants, and animals (Luhtala & Parker, 2010). Recently, RNase T2 was determined to be a new BECR family, characterized by a circularly permutated version of the BECR fold (Li et al., 2023). Most members of the RNase T2 family have been reported to be secreted from cells or localized in internal compartments such as the vacuole or lysosome, suggesting their functional diversity (Yue et al., 2023). Previous studies reported that RNase T2 performs some important roles during evolutionary adaptation and potentially functions as a new type of effector, indicating a possible role of RNase T2 proteins in fungal virulence (Mukherjee et al., 2020; Yang et al., 2021). However, there are few studies about the involvement of RNase T2 in host–pathogen interactions, and its biological functions have remained unclear. In this study, we identified a secreted RNase protein with the RNase T2 domain, belonging to the RNase T2 family, in the Foc TR4 secretome. It was characterized as a classically secreted protein with a signal peptide, without any GPI‐anchor site and transmembrane domain. FocRnt2 is highly conserved among phytopathogenic *Fusarium* (Figure S1), suggesting that it may play a key role in fungal biology. Our results also showed that FocRnt2 protein was localized in both the nucleus and cytoplasm of *N*. *benthamiana* leaves using *Agrobacterium*‐mediated transient expression (Figure 4). Similar to our results, FoRnt2, which is secreted from *F*. *oxysporum* f. sp. *lycopersici* and belongs to the RNase T2 family, is also present in the cell cytoplasm and nucleus in *N*. *benthamiana* (Qian et al., 2022). However, further investigations are needed to find the possible target proteins of the FocRnt2 protein and the possible regulatory mechanism in host plants.

The function of FocRnt2 was investigated using *Agrobacterium*‐mediated transient expression in leaves of the non‐host *N*. *benthamiana*. Our results showed that FocRnt2 with or without the signal peptide could not induce cell death and ROS accumulation in *N*. *benthamiana* leaves (Figure S2). Similar to our results, transient expression of FoRnt2 from *F*. *oxysporum* f. sp. *lycopersici* did not induce cell death (Qian et al., 2022). Furthermore, we noticed that FocRnt2 protein with or without the signal peptide could inhibit both BAX‐induced cell death and ROS accumulation in *N*. *benthamiana* (Figure 3), suggesting that the signal peptide of FocRnt2 may not be necessary for its cell death‐suppressing ability. It is well documented that the effectors from phytopathogenic fungi play key roles in suppressing plant defences and promoting fungal infection (Shu et al., 2022). Several effectors that cause cell death in leaves of *N*. *benthamiana* have been identified in previous studies. For example, the effector FocM35_1 suppresses BAX‐induced cell death in *N*. *benthamiana* and plays an important role in full virulence in Foc TR4 (Zhang et al., 2021). A cupin type‐1 domain‐containing protein, FoCupin1, was found to be an essential virulence effector of Foc TR4 and can suppress BAX‐induced cell death in *N*. *benthamiana* (Yan et al., 2022). Furthermore, FocRnt2 significantly down‐regulated the expression of defence‐related genes (*NbPR5*, *NbPR4*, *NbLOX*, and *NbEIN2*) in *N*. *benthamiana* (Figure 3). Therefore, these results suggest that FocRnt2 may suppress plant immune responses, thus enhancing fungal virulence.

Effectors are often highly expressed during invasion or after induction by the host plants. In this study, the transcript level of *FocRnt2* was significantly up‐regulated during early stages of fungal infection in planta or after induction by banana root extracts in vitro, indicating that FocRnt2 may play a vital role in fungal virulence (Figure 2). To further characterize the function of FocRnt2, we deleted the *FocRnt2* gene and found that the *FocRnt2* deletion reduced Foc TR4 pathogenicity to banana (Figure 6) but had little effect on hyphal growth, conidiation, and sensitivity to cell wall, osmotic, and oxidative stresses (Figure 5). Consistent with our results, previous studies showed that RNase T2 proteins also probably contribute to fungal virulence. For example, Nuc1 and Nuc2 of *U. maydis*, which belong to the RNase T2 family, are involved in fungal virulence during maize infection (Mukherjee et al., 2020). FoRnt2, a secretory RNase T2 protein of *F*. *oxysporum* f. sp. *lycopersici*, is required for the full virulence and can promote fungal pathogen infection in plants (Qian et al., 2022). In this study, we also observed that FocRnt2 can regulate the biosynthesis of FA in Foc TR4 (Figure 6d,e), which provides new evidence that the RNase T2 protein may play multiple different roles in fungal virulence. Histopathological observation of the fungal infection process showed that *FocRnt2* deletion compromised fungal colonization and expansion in the infected roots (Figure 7). However, the underlying mechanisms of the FocRnt2 protein regulating FA production and fungal virulence are still further studied.

Fungal pathogens secrete a repertoire of effectors that modulate host immunity and facilitate infection (Todd et al., 2022). Several effector proteins have been reported in Foc4, including SIX proteins, FocCP1, FocM35_1, Fosp9, and FoCupin1 (An et al., 2019; Guo et al., 2022; Liu et al., 2019; Yan et al., 2022; Zhang et al., 2021). Most of these reported effectors were required for Foc4 pathogenesis (He et al., 2021). Despite this, research on the effectors of Foc4 is generally scarce. In this study, we identified a novel effector, FocRnt2, from Foc TR4. Similar to previously reported effectors, the FocRnt2 deletion decreased toxin production, compromised fungal infection, up‐regulated expression of defence‐related genes in banana, and reduced virulence of Foc TR4 to banana plants. Moreover, transient expression of FocRnt2 in *N*. *benthamiana* suppressed host immunity and ROS accumulation. The expression of the *FocRnt2* gene was significantly induced during fungal infection. Together with the above studies, our results provide new evidence that FocRnt2 can function as a new effector to suppress plant immunity, thereby promote fungal infection, and is required for the full virulence of Foc TR4.

## 4 EXPERIMENTAL PROCEDURES

### 4.1 Plant and fungus

Foc TR4 strain DZ1 was used as the WT strain in the study (Qin et al., 2017). The Foc TR4‐susceptible banana cultivar Brazilian (AAA group, Cavendish) and *N. benthamiana* were grown in a greenhouse at 25 ± 1°C, 70%–80% relative humidity with a 12 h photoperiod. Banana seedlings at fully developed four‐leaf stage were used for all experiments. Five‐week‐old *N*. *benthamiana* was used for infiltration and transformation assays. PDA was used as regular growth medium, while CD medium was used for conidiation assays of Foc TR4 (Li et al., 2014). NCMB liquid medium was used to mimic Foc–banana interaction (He et al., 2021). PDA was supplemented with sorbitol (1.2 M), calcofluor white (CFW, 100 μg/mL), sodium dodecyl sulphate (SDS, 0.05%), hydrogen peroxide (H_2_O_2_, 300 mM), or Congo red (CR, 200 μg/mL) for fungal stress response assays. To determine fungal growth rates in liquid medium, potato dextrose broth was inoculated with freshly prepared conidial suspension at a final concentration of 10^5^ conidia/mL and then cultured at 28°C for different time intervals (1–5 days).

### 4.2 Bioinformatics analysis

The multiple sequence alignment of FocRnt2 (gene name: *FOIG_10760*) and its homologues in different formae speciales of *F*. *oxysporum* and other phytopathogens was generated using the ClustalX v. 2.1 program. Phylogenetic trees were constructed using MEGA X software with the neighbour‐joining method and 1000 bootstraps (Kumar et al., 2018). Five bioinformatics packages, SignalP v. 5.0, TargetP v. 1.1, TMHMM v. 2.0, WoLF PSORT, and big‐PI predictor, were applied for characterizing the FocRnt2 protein as described (He et al., 2021). The conserved domains of FocRnt2 were identified using the Pfam database (http://pfam.xfam.org) (Mistry et al., 2021), and the candidate effector analysis was performed using EffectorP v. 3.0 (http://effectorp.csiro.au/) (Sperschneider et al., 2018).

### 4.3 Yeast signal sequence trap assay

Functional evaluation of the signal peptide (SP) of FocRnt2 was performed with a yeast signal sequence trap assay (Yin et al., 2018). The pSUC2 vector contains a truncated invertase gene lacking methionine (Met) and SP. The SP sequence of FocRnt2 was cloned into the pSUC2 vector to generate the pSUC2:FocRnt2 construct. The SP of *Phytophthora sojae* effector Avr1b was cloned into the pSUC2 vector to generate the pSUC2:Avr1b construct as a positive control. These derived vectors were transformed into the yeast strain YTK12. The yeast strain YTK12 and YTK12 carrying pSUC2 empty vector were used as negative controls. These YTK12 strains were then grown on YPDA (a complete medium), CMD−W medium (lacking Trp), and YPRAA medium to detect invertase secretion. The invertase activity of these yeast strains was confirmed by the reduction of 2,3,5‐triphenyltetrazolium chloride (TTC) to the insoluble red‐coloured triphenylformazan. All the experiments were repeated three times.

### 4.4 RNA extraction and RT‐qPCR analysis

Total RNA from Foc TR4, banana, and *N*. *benthamiana* was extracted using the Fungal RNA kit (Omega) and Plant RNA Kit (Omega) according to the manufacturer's instructions, respectively. RT‐qPCR was subsequently conducted using the SYBR Premix Ex Taq Kit (TaKaRa), following the manufacturer's instructions. For Foc TR4, banana, and *N. benthamiana*, *FocEF1a*, *MaActin*, and *NbEF1a* were used as internal references, respectively. The primers used for RT‐qPCR were listed in Table S1. Relative transcript levels for each gene were determined as previously described (Livak & Schmittgen, 2001). All experiments were repeated three times.

### 4.5 Ribonuclease activity assays

The coding sequence of the *FocRnt2* gene was cloned into the expression vector pET‐32a. The recombinant vectors or empty vectors were transformed into *E*. *coli* Rosetta (DE3). IPTG (0.1 mM) was used to induce the expression of the recombinant proteins for 12 h at 16°C. The *E*. *coli* cells were collected by centrifugation and sonication. Recombinant proteins were purified using a BeyoGold His‐tag Purification Resin (Beyotime). Protein concentration was determined using the Bradford method, with bovine serum albumen (BSA) as the standard (Bradford, 1976). The purity of the proteins was determined by SDS‐PAGE, which was stained with the Coomassie brilliant blue G‐250 (CBB) staining method. The RNase activity of FocRnt2 was conducted by incubation with total RNA from banana roots in an in vitro assay (Yang et al., 2021). Total RNA was incubated with the recombinant protein Trx‐His‐FoRnt2 at 25°C for 45 min. RNase A was used as a positive control, while the tag protein Trx‐His was used as a negative control. All the experiments were repeated three times.

### 4.6 Agroinfiltration assays

FocRnt2 with the signal peptide (SPFocRnt2) and without the signal peptide (NSPFocRnt2) was cloned in the pBI121 vector. The recombinant constructs were introduced into *A. tumefaciens* GV3101 by electroporation, subsequently infiltrated into the leaves of *N*. *benthamiana* as described by Ma et al. (2012). The pBI121 vector containing BAX protein and the translationally controlled tumour protein (TCTP) were used as positive and negative controls, respectively (Hoepflinger et al., 2013; Lacomme & Cruz, 1999). The inoculated leaves were photographed 4 days after infiltration. All experiments were repeated three times.

### 4.7 Subcellular localization assay

SPFocRnt2 or NSPFocRnt2 was separately fused to green fluorescent protein (GFP) at its C‐terminus in the pEarley103 vector according to the Gateway protocol for LR recombination reaction (Invitrogen). The recombinant constructs were transformed into *N*. *benthamiana* leaves separately using *Agrobacterium*‐mediated transformation as described previously (Gawehns et al., 2014). H2B‐RFP (red fluorescent protein) transgenic *N*. *benthamiana* was used as a positive control. After 2 days, leaves were harvested for fluorescence observation using an Axiovert 200 M microscope equipped with a LSM 780 META system (Zeiss). Images were acquired and processed using LSM 710 AIM v. 4.2 SP1 software (Zeiss).

### 4.8 Gene deletion and complementation

The knockout vector for *FocRnt2* was constructed by a homologous recombination approach, according to Yan et al. (2022). Briefly, the left and right flanking sequences of *FocRnt2* were inserted into the pCT74 vector at the XhoI‐KpnI and EcoRI‐SpeI sites, respectively. Polyethylene glycol (PEG)‐mediated transformation protoplast transformation was performed as described previously (Wang et al., 2020). The candidate deletion mutants were screened by PCR, Southern blot, and RT‐qPCR analysis. Gene complementation was further performed by transformation of the *FocRnt2* deletion mutant with the pCTZN vector, which contained the entire coding region of *FocRnt2* with its native promoter and terminator. The putative complementation strains were selected by resistance to zeocin, PCR, and RT‐qPCR analysis. Primers in the construction and screening of different strains were listed in Table S1.

### 4.9 Stress sensitivity assays

To test *FocRnt2* mutants responses against various stresses, different fungal strains were cultured on PDA supplemented with NaCl, SDS, sorbitol, CR, CFW, or H_2_O_2_ for 5 days at 28°C, as described by Yan et al. (2022). Cellophane penetration was determined as described by Dai et al. (2016). All the experiments were repeated at least three times.

### 4.10 Pathogenicity tests

Fungal inoculation was performed as described (Dong et al., 2019). Briefly, banana plantlets were soaked in conidial suspension (10^5^ conidia/mL) for 30 min, then planted in potting soil and grown in a greenhouse at 25 ± 1°C. The disease symptoms were observed 4 weeks after inoculation, and the disease index was calculated according to An et al. (2019). To determine the fungal biomass, banana roots were collected at 24, 48, and 72 h after inoculation. qPCR was conducted, and the relative fungal biomass was estimated by equation 2^[*C*t(*MaActin*)−*C*t(*FoEF1a*)]^ as described (Dai et al., 2016). All the experiments were repeated three times.

For confocal laser scanning microscope observation, the GFP‐tagged Foc TR4 strains, Δ*FocRnt2* and ΔF*ocRnt2*‐com strains, were used to observe the infection process. After inoculation, the infected plantlet roots were harvested at 1, 2, 3, 4, and 5 days. Microscopic observation was carried out under a confocal laser‐scanning microscope (LSM 780; Zeiss) equipped with filter blocks. Excitation/ emission wavelengths were set as 488/520 nm for GFP and 543/590 nm for autofluorescence of plant tissues, respectively. Nine plants were prepared for each time point, and each experiment was repeated three times.

### 4.11 Fusaric acid determination

Fusaric acid determination was conducted as described by Yan et al. (2022). Briefly, fungal mycelium was cultured in CD medium for 9 days at 30°C. The culture solution was sterilized at 121°C for 18 min and ultrasonicated for 10 min, then filtered with double gauze. After centrifugation at 3822 *g* for 30 min, the supernatant was extracted with an equal volume of ethyl acetate. The extract was revolved to dryness at 45°C, redissolved with anhydrous ethanol. FA contents were detected by measuring the absorbance at 268 nm. The experiments were repeated three times.

### 4.12 Statistical analysis

Statistical analyses were carried out using SPSS v. 14.0 software. To determine the significant difference among group means, the repeated measurement was given as means ± *SE*. Multiple differences among means were evaluated using Duncan's multiple range tests at a 5% probability level.

## CONFLICT OF INTEREST STATEMENT

The authors declare no competing interests.

## Supporting information


Figure S1.

Figure S2.

Figure S3.

Figure S4.

Figure S5.

Table S1.

## Data Availability

The data that support the findings of this study are available in the supplementary material of this article.
