# Supplementary material for: A ribonuclease T2 protein FocRnt2 contributes to the virulence of Fusarium oxysporum f. sp. cubense tropical race 4
Source: Mol Plant Pathol. 2024 Aug 8;25(8):e13502. doi: 10.1111/mpp.13502 (PMC11310096; doi:10.1111/mpp.13502)
Supplement: Supplementary file 1 — Figure S1. [file MPP-25-e13502-s001.pdf]

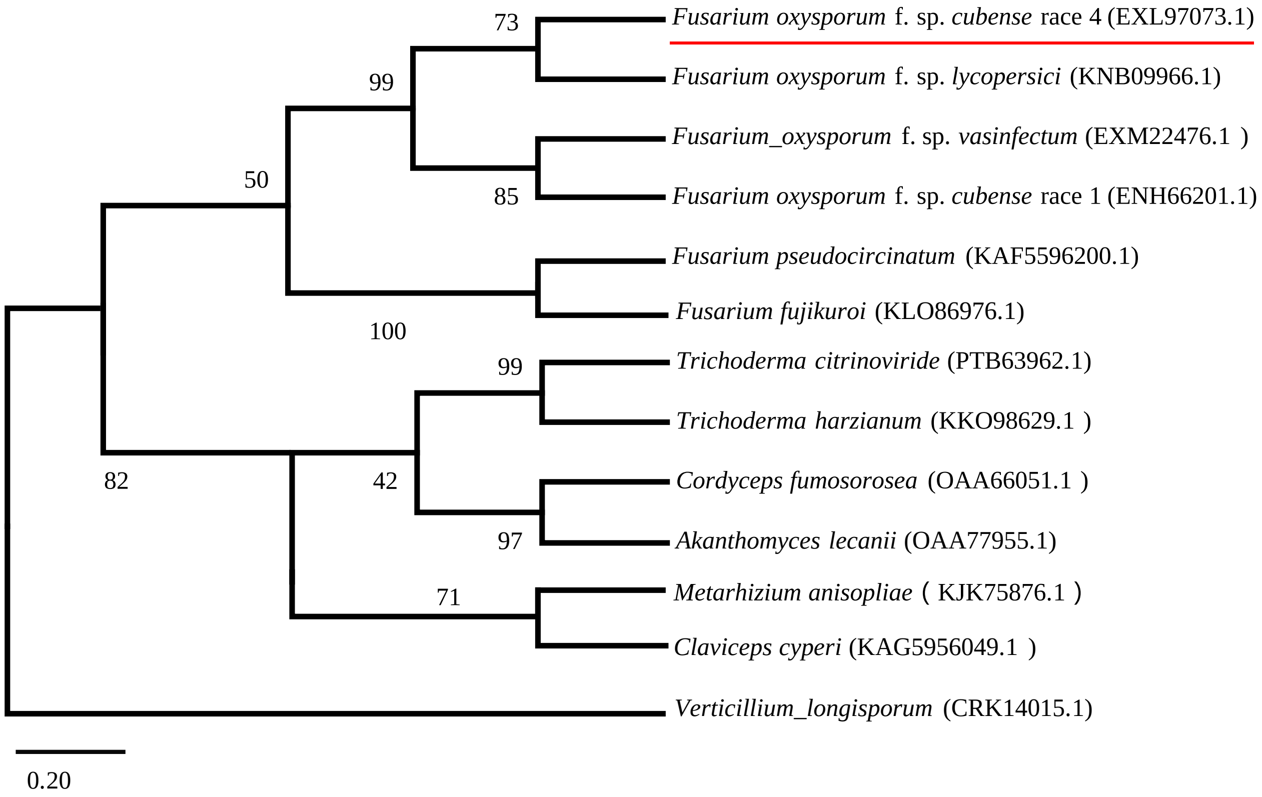

Supplemental Figure S1: Phylogenetic analysis of FocRnt2 protein from Foc TR4. The maximum likelihood tree was constructed using the sequence of FocRnt2 and its orthologous proteins from sixteen different fungal pathogens.
