# Supplementary material for: A ribonuclease T2 protein FocRnt2 contributes to the virulence of Fusarium oxysporum f. sp. cubense tropical race 4
Source: Mol Plant Pathol. 2024 Aug 8;25(8):e13502. doi: 10.1111/mpp.13502 (PMC11310096; doi:10.1111/mpp.13502)
Supplement: Supplementary file 2 — Figure S2. [file MPP-25-e13502-s002.pdf]

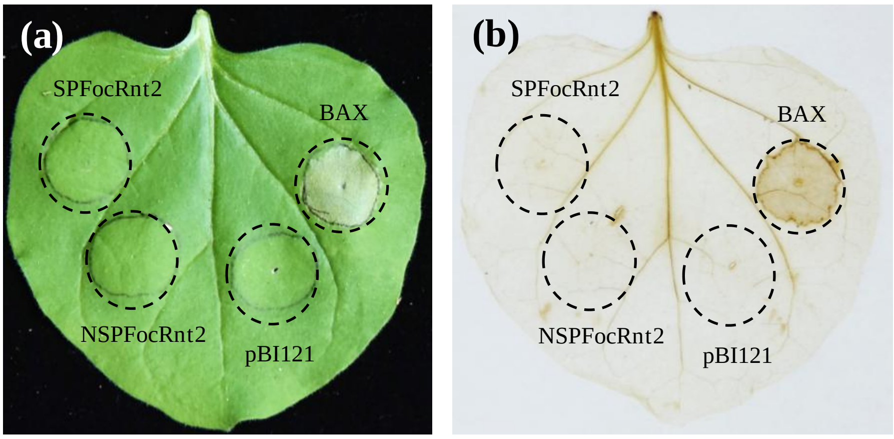

Supplemental Figure S2: FocRnt2 could not induce the cell death (a) and ROS accumulation (b) in *N. benthamiana*. *N. benthamiana* leaves infiltrated with *A. tumefaciens* expressing SPFocRnt2, NSPFocRnt2, BAX (as positive control), or pBI121 empty vector (as negative control). The cell death was photographed 3-4 d after infiltration. ROS accumulation in (a) was detected by DAB staining (b).
