# Supplementary material for: A ribonuclease T2 protein FocRnt2 contributes to the virulence of Fusarium oxysporum f. sp. cubense tropical race 4
Source: Mol Plant Pathol. 2024 Aug 8;25(8):e13502. doi: 10.1111/mpp.13502 (PMC11310096; doi:10.1111/mpp.13502)
Supplement: Supplementary file 3 — Figure S3. [file MPP-25-e13502-s006.pdf]

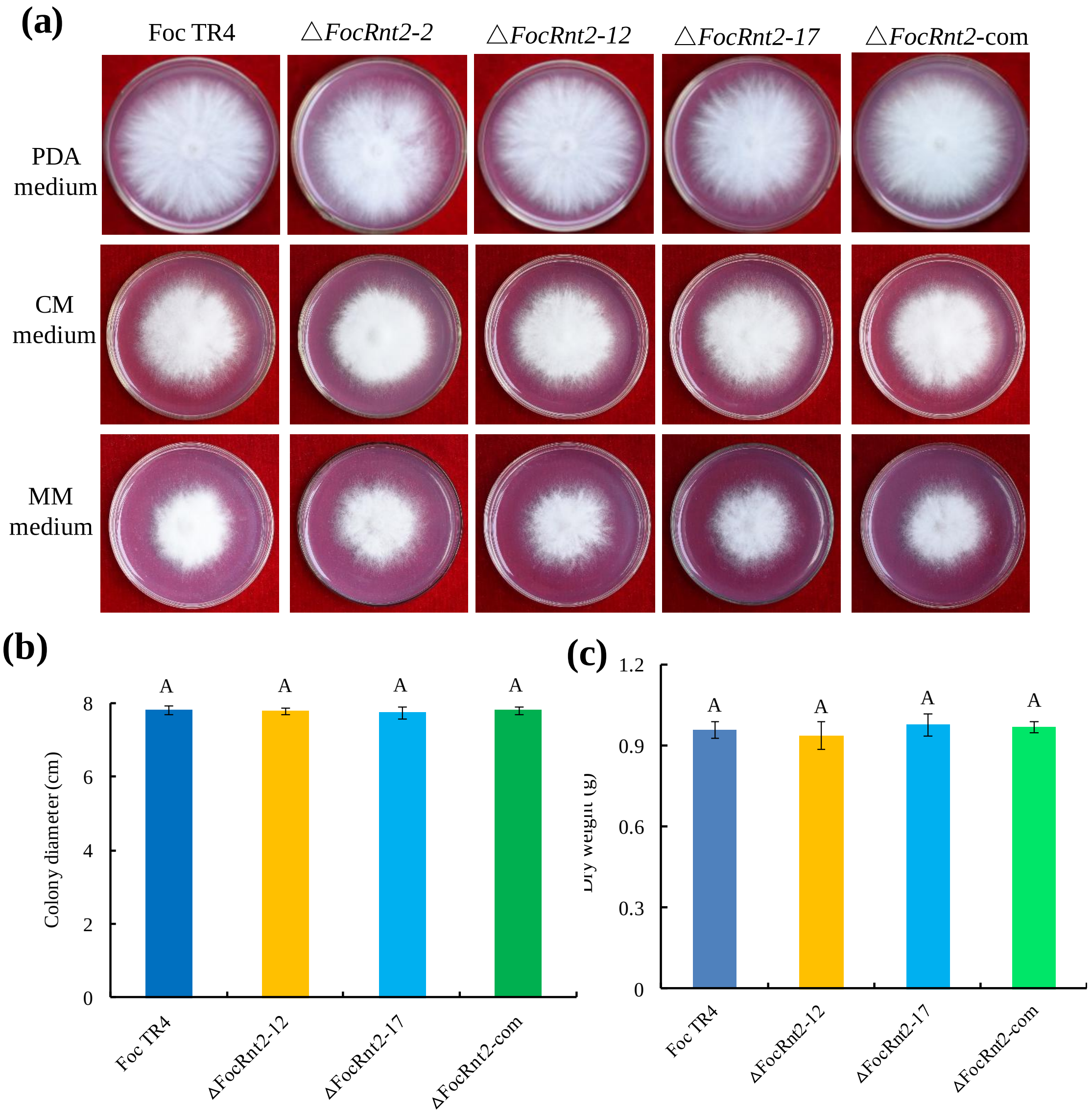

Supplemental Figure S3: Colony morphology (a), grow rate (b) and dry weight (c) of *FocRnt2* deletion mutants and the complementation strain. Foc TR4, the wide-type strain; Δ*FocRnt2-2*, Δ*FocRnt2-12* and Δ*FocRnt2-17*, *FocRnt2* deletion mutants; Δ*FocRnt2-com*, *FocRnt2* complementation strain. Images were taken at 5 dpi. Values are the means (±SE) based on three independent experiments and bars indicate standard deviations. The letters above the bars indicate significant difference at 0.05 level using Duncan's multiple range test.
