# Supplementary material for: A ribonuclease T2 protein FocRnt2 contributes to the virulence of Fusarium oxysporum f. sp. cubense tropical race 4
Source: Mol Plant Pathol. 2024 Aug 8;25(8):e13502. doi: 10.1111/mpp.13502 (PMC11310096; doi:10.1111/mpp.13502)
Supplement: Supplementary file 4 — Figure S4. [file MPP-25-e13502-s005.pdf]

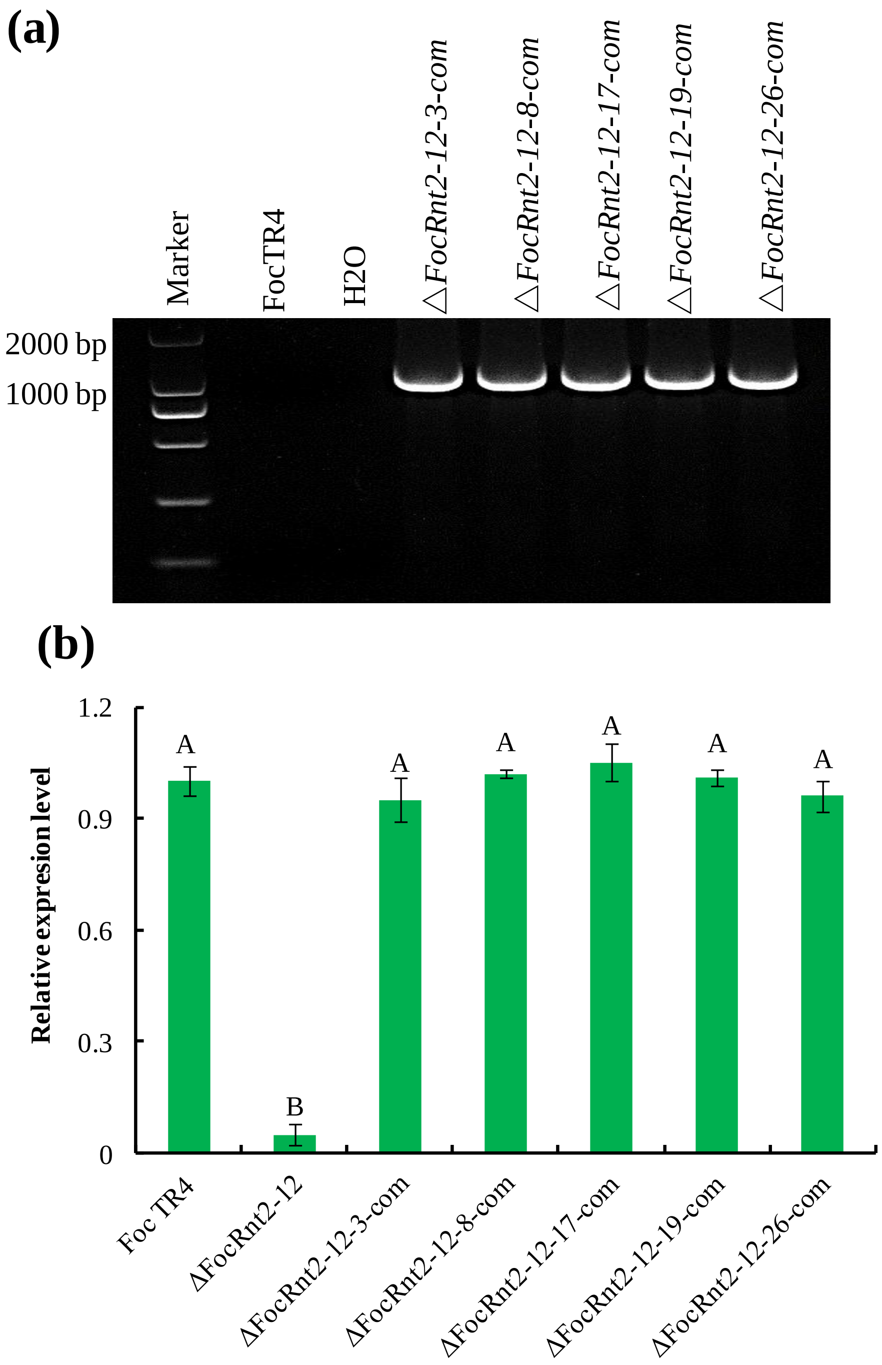

Supplemental Figure S4: PCR (a) and RT-qPCR (b) confirmation of *FocRnt2* complementation strains. The *FocRnt2* gene fragment was used as a probe. Foc TR4, the wide-type strain; Δ*FocRnt2-12*, *FocRnt2* deletion mutant; *FocRnt2-12-3-com*, *FocRnt2-12-8-com*, *FocRnt2-12-17-com*, *FocRnt2-12-19-com* and *FocRnt2-12-26-com*, *FocRnt2* complementation strains.
