# Supplementary material for: A ribonuclease T2 protein FocRnt2 contributes to the virulence of Fusarium oxysporum f. sp. cubense tropical race 4
Source: Mol Plant Pathol. 2024 Aug 8;25(8):e13502. doi: 10.1111/mpp.13502 (PMC11310096; doi:10.1111/mpp.13502)
Supplement: Supplementary file 5 — Figure S5. [file MPP-25-e13502-s003.pdf]

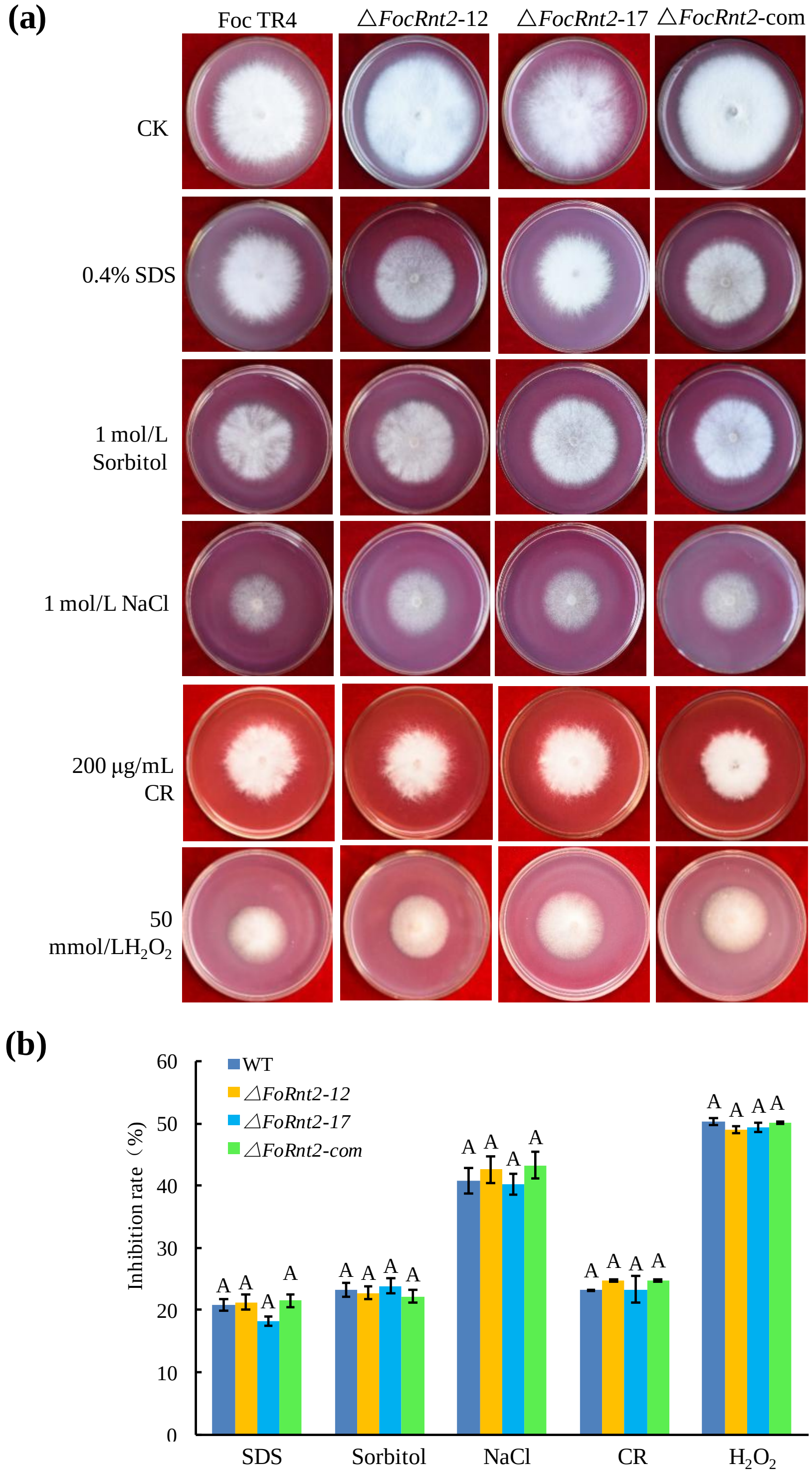

Supplemental Figure S5: Colony morphology (a) and growth inhibition rate (b) of *FocRnt2* gene deletion mutants with different stresses. Photographs were taken 5 d after incubation. Growth inhibition =(diameter of untreated strain - diameter of treated strain) / (diameter of untreated strain) × 100%. Foc TR4, the wide-type strain; Δ*FocRnt2-12* and Δ*FocRnt2-17*, *FocRnt2* deletion mutants; Δ*FocRnt2*-com, *FocRnt2* complementation strain. Values are the means (±SE) based on three independent experiments and bars indicate standard deviations. The same letter are not significantly different at p<0.05 by Duncan' s multiple range test.
