# Supplementary material for: A ribonuclease T2 protein FocRnt2 contributes to the virulence of Fusarium oxysporum f. sp. cubense tropical race 4
Source: Mol Plant Pathol. 2024 Aug 8;25(8):e13502. doi: 10.1111/mpp.13502 (PMC11310096; doi:10.1111/mpp.13502)
Supplement: Supplementary file 6 — Table S1. [file MPP-25-e13502-s004.pdf]

Supplemental Table S1: Primers used in this study

Primer name	Primer Sequence (5'-3')	Reference
Construction and confirmation of *FocRnt2* deletion and complementation mutants		
FocRnt2-upF	GGGGTACCTTGGCGATGGTGCTGAGTGG	Present work
FocRnt2-upR	CCGCTCGAGTGTTGGTTCCGGGGCTGATA	Present work
FocRnt2-downF	GGAATTCCATCGTTCTTTTCAACGTTATC	Present work
FocRnt2-downR	GACTAGTTAGGAGCATCACATTCGTAA	Present work
hph-F	TGCTGCTCCATACAAGCCAA	Present work
hph-R	GACATTGGGGAGTTCAGCGA	Present work
FocRnt2-F	ATGCTCAGTTACGGGTTGCG	Present work
FocRnt2-R	CAAGTAAAGTACGTGATTACATACGG	Present work
FocRnt2-comF	GGGGTACCGGAGAAGAAACCGAGATTGTAATT	Present work
FocRnt2-comR	ACGCGTCGACCGAAACATGCCATAGTGGAGAT	Present work
FocRnt2 probe-F	ATGCTCAGTTACGGGTTGCG	Present work
FocRnt2 probe-R	CAAGTAAAGTACGTGATTACATACGG	Present work
hph-porobe -F	TGCTGCTCCATACAAGCCAA	Present work
hph-porobe -R	GACATTGGGGAGTTCAGCGA	Present work
Construction of transient expression vector		
pENTR-SP*FocRnt2*-F	ccgcggccgccccсttcacc ATGTCTTCGTTTCAGATTTTGTC	Present work
pENTR-SP*FocRnt2*-R	gggtcggcgcgcccaccctt AAGGTCCTCCACATCCGCA	Present work
pENTR-NSP*FocRnt2*-F	ccgcggccgccccсttcacc ATGGCCGGTCCCAGCGCTACA	Present work
pENTR-NSP*FocRnt2*-R	gggtcggcgcgcccaccctt AAGGTCCTCCACATCCGCA	Present work
RT-qPCR analysis for *FocRnt2* and fusaric acid in Foc TR4		
q*FocRnt2*-F	TGGTAGTCGCCGAGGCTCAT	Present work
q*FocRnt2*-R	TGGTGTCGCAGTGGGTAGGG	Present work
q*FoEF1α*-F	GCTGGTGACTCCAAGAACGA	Liu at al., 2019
q*FoEF1α*-R	CATCTTGACGATGGCGGAGT	Liu at al., 2019
FOIG_16450-F	CATCAACAGTCCCGCCAGTG	Ding at al., 2020
FOIG_16450-R	CGGAGTTTGCGAGCGAAGATA	Ding at al., 2020
FOIG_16451-F	CCACAGCACTGCCGAAAATG	Ding at al., 2020
FOIG_16451-R	TGACGAAGAAGCCGTGAGACA	Ding at al., 2020
FOIG_16452-F	GCAAAGCAAAGGACAAAATGG	Ding at al., 2020
FOIG_16452-R	GCAGCAGCCTCGTGGAAGAA	Ding at al., 2020
FOIG_16453-F	CGAGAAGCCCCAGACACCAT	Ding at al., 2020
FOIG_16453-R	TCCCCAAGCCCAACTACAGC	Ding at al., 2020
FOIG_16454-F	TGCTACATCGCCCTCACCAAC	Ding at al., 2020
FOIG_16454-R	CACAAGCGTAGGCTGCTCAAT	Ding at al., 2020
DNA-based qPCR analysis of fungal biomass		
q*FoEF1α*-F	GCTGGTGACTCCAAGAACGA	Liu at al., 2019
q*FoEF1α*-R	CATCTTGACGATGGCGGAGT	Liu at al., 2019

q*MaActin*-F	TGTTGCATCCTGGTACTGCT	Liu at al., 2019
q*MaActin*-R	GGCTTTCTTGCACTGGTACAC	Liu at al., 2019
RT-qPCR analysis of defense-related genes in tobacco and banana		
q*NbEF1α*-F	GGTTAAGATGATGCCGACCAAG	Zhang at al., 2017
q*NbEF1α*-R	CGCCAGTTGGGTCCTTCTTG	Zhang at al., 2017
q*NbPR5*-F	GGGCCAATCTTGGAGCATTA	Present work
q*NbPR5*-R	CAGTCTCCAGTCTCACAATTACC	Present work
q*NbPR4*-F	GGCCAAGATTCCTGTGGTAGAT	Zhang at al., 2017
q*NbPR4*-R	CACTGTTGTTTGAGTTCCTGTTCCT	Zhang at al., 2017
q*NbLOX*-F	AAAACCTATGCCTCAAGAAC	Zhang at al., 2017
q*NbLOX*-R	ACTGCTGCATAGGCTTTGG	Zhang at al., 2017
q*NbEIN2*-F	CGTCAACTATGCTGAACCATTTG	Present work
q*Nb EIN2*-R	ACGGGCTGCATGGAATTAT	Present work
q*MaPR1*-F	AGGACAACGAGGGGGAGATA	Niu at al., 2018
q*MaPR1*-R	TACGGGTAGGCTGATGGGTT	Niu at al., 2018
q*MaNPR1*-F	GGAGATCCACAAGTAGGTGAAGC	Dalio at al., 2020
q*MaNPR1*-R	AGTCTTGCCAGAGCAACTCG	Dalio at al., 2020
q*MaPR3*-F	GTCACCACCAACATCATCAA	Dalio at al., 2020
q*MaPR3*-R	CCAGCAAGTCGCAGTACCTC	Dalio at al., 2020
q*MaERF1*-F	CCCAAATGTTGGTCCGTTTC	Dalio at al., 2020
q*MaERF1*-R	TCGCTGTCTTCCACGATTCA	Dalio at al., 2020
q*MaACC*-F	GATGCTGCACATCGGCTAGT	Dalio at al., 2020
q*MaACC*-R	GCCACCTGAATACGGCAGAC	Dalio at al., 2020
q*MaMYC2*-F	CGGATCTACCGACGTGGTCT	Dalio at al., 2020
q*MaMYC2*-R	AGCGTCCGGAGAGCTAAAGT	Dalio at al., 2020
q*MaActin*-F	TGTTGCATCCTGGTACTGCT	Liu at al., 2019
q*MaActin*-R	GGCTTTCTTGCACTGGTACAC	Liu at al., 2019

Note: The lowercase letters of the primer sequence are the linker sequence on the pENTR-TOPO vector.

References for Supplemental Materials

Dalio, R. J. D., Maximo, H. J., Roma-Almeida, R., Barretta, J. N., Jose, E. M., Vitti, A. J. *et al*. (2020) Tea Tree Oil Induces Systemic Resistance against Fusarium wilt in Banana and Xanthomonas Infection in Tomato Plants. Plants, 9 (9), 1137.

Ding, Z., Xu, T., Zhu, W., Li, L., Fu, Q., (2020) A MADS-box transcription factor FoRlm1 regulates aerial hyphal growth, oxidative stress, cell wall biosynthesis and virulence in *Fusarium oxysporum* f. sp. *cubense*. Fungal Biology, 124 (3-4), 183-193.

Liu, S., Wu, B., Yang, J., Bi, F., Dong, T., Yang, Q. *et al*., (2019) A Cerato-Platanin Family Protein FocCP1 Is Essential for the Penetration and Virulence of *Fusarium oxysporum* f. sp. *cubense* Tropical Race 4. International Journal of

Molecular Sciences, 20 (15), 3785.

Niu, Y., Hu, B., Li, X., Chen, H., Takac, T., Samaj, J. *et al*., (2018) Comparative Digital Gene Expression Analysis of Tissue-Cultured Plantlets of Highly Resistant and Susceptible Banana Cultivarsin Response to *Fusarium oxysporum*. International Journal of Molecular Sciences, 19 (2), 350.

Zhang, L., Ni, H., Du, X., Wang, S., Ma, X.-W., Nuernberger, T. *et al*., (2017) The Verticillium-specific protein VdSCP7 localizes to the plant nucleus and modulates immunity to fungal infections. New Phytologist, 215 (1), 368-381.
